# TYPES OF ATTENDEES IN COMMUNITY SPACE IN JAPAN: TOWARD DESIGNING AN ENVIRONMENT FOR SOCIAL PARTICIPATION

**DOI:** 10.1093/geroni/igac059.2002

**Published:** 2022-12-20

**Authors:** Mai Takase, Ryogo Ogino, Ryoichi Nitanai, Riko Nakayama, Hongjik Kim, Neo Kazembe, Jun Goto, Katsuya Iijima

**Affiliations:** The University of Tokyo, Tokyo, Tokyo, Japan; Saga University, Saga, Saga, Japan; The University of Tokyo, Tokyo, Tokyo, Japan; The University of Tokyo, Tokyo, Tokyo, Japan; The University of Tokyo, Tokyo, Tokyo, Japan; The University of Tokyo, Tokyo, Tokyo, Japan; Tokai University, Hiratsuka, Tokyo, Japan; The University of Tokyo, Tokyo, Tokyo, Japan

## Abstract

**Introduction:**

Designing community space for social participation of older adults is essential for healthy aging. The behavioral characteristics of the attendees have much to offer as they relate to social settings designed in the space. This study aims to elucidate the basic typology of the attendees considering their motivation for attendance and state of social connection.

**Methods:**

Semi-structured interviews targeting attendees of Chiiki-Katsudokan, a space designed for social participation in a larger aging estate in Japan, were conducted in December 2021 (N=16, 16% male). Attendees were asked about their reasons for attending events at Chiiki-Katsudokan, interaction levels with other attendees, and levels of social participation during the COVID-19 pandemic.

**Results:**

Based on the interviews, attendees were classified into three types: Seeker, Hobbyist, and Socializer. First, Seekers (n=3) used Chiiki-Katsudokan as the primary means of preventing social isolation. Amongst the three types, the social interaction level of Seekers was the lightest, and the opportunity decreased when Chiiki-Katsudokan closed during the quarantine. Second, the main motivation for Hobbyists (n=5) was the event contents which matched their interests. Hobbyists were also likely to engage in hobby networks, which continued privately during the pandemic. Finally, Socializers (n=8) attended Chiiki-Katsudokan to communicate with friends. Many were initially socially active and engaged in social interaction outside Chiiki-Katsudokan, e.g., teatime with friends, during the quarantine.

**Conclusion:**

This study suggested that designing community space for social participation requires defining the varying levels of engagement and expecting relationships outside the space based on the behavioral characteristics of the attendees.

